# Risk factors for episiotomy during vaginal birth: A systematic review and meta-analysis

**DOI:** 10.1097/MD.0000000000049662

**Published:** 2026-07-10

**Authors:** Xuemei He, Demei Lu, Ruifeng Ou, Jinping Feng

**Affiliations:** a Department of Obstetrics, The Affiliated FoShan Women and Children Hospital, Guangdong Medical University, FoShan, China; b Department of Nursing, The Affiliated Foshan Women and Children Hospital, Guangdong Medical University, Foshan, China.

**Keywords:** episiotomy, meta-analysis, pregnant, risk factors, vaginal delivery

## Abstract

**Background:**

Despite the numerous harms associated with episiotomy during vaginal birth, the risk factors for this procedure remain a topic of controversy. This study aimed to identify the potential risk factors for episiotomy during vaginal birth.

**Methods:**

A systematic search was conducted across 7 databases, including PubMed/MedLine, Embase, Cochrane, Web of Science, Chinese Knowledge Infrastructure (CNKI), and Wanfang, up to January 2025. Statistical analyses, including heterogeneity tests, sensitivity analysis, and publication bias assessment, were conducted using Stata 18.0 and Review Manager 5.4.

**Results:**

A total of 16 articles were included in this meta-analysis, comprising a sample size of 17,502 individuals. Newborn weight (odds ratio [OR] = 2.393; 95% confidence interval [CI] = 1.627–3.520; *P* < .001) and oxytocin use (OR = 2.341; 95% CI = 1.652–3.318; *P* < .001) were identified as significant risk factors for episiotomy. In contrast, maternal age (OR = 0.978; 95% CI = 0.922–1.038; *P* = .466), the type of childbirth attendant (OR = 0.920; 95% CI = 0.569–1.488; *P* = .733), and the duration of the second stage of labor (OR = 1.299; 95% CI = 0.781–2.162; *P* = .314) were not statistically significant risk factors for episiotomy.

**Conclusion:**

High newborn weight and oxytocin use are associated with higher rates of episiotomy. However, other potential risk factors are still controversial due to a lack of sufficient evidence, highlighting the need for more rigorous studies to strengthen the evidence base.

## 1. Introduction

Episiotomy, which involves an incision in the perineum to facilitate childbirth, has historically been a common practice aimed at protecting the pelvic floor and reducing fetal injury.^[1,2]^ However, research has shown that episiotomy is associated with various complications, including increased blood loss, perineal pain, severe lacerations, a higher need for suturing, unresolved benefits for both mother and infant, negative birth experiences, elevated rates of infection and dehiscence, and long-term issues such as urinary incontinence, sexual dysfunction, and an increased risk of perineal lacerations in future deliveries.^[3-6]^

The World Health Organization (WHO) recommends that routine episiotomy should be avoided, advising that the incidence should not exceed 10%. Despite its widespread application, many clinical settings report episiotomy rates that far surpass this threshold.^[7]^ A comprehensive review from the Cochrane Library indicates that selective episiotomy, conducted during spontaneous labor without instrumentation, results in 30% fewer instances of severe vaginal trauma compared to regular episiotomy.^[8]^ Consequently, doubts have arisen regarding the conventional practice of episiotomy, as research suggests that the associated risks may not justify its benefits.^[9]^ Recent findings underscore the necessity for ongoing medical education and initiatives aimed at educating healthcare professionals involved in childbirth to mitigate the procedure’s prevalence.^[10-12]^ Additionally, it is essential to document the factors influencing the use of episiotomy and to monitor the gradual reduction in its application following the implementation of policies designed to limit its use.

Currently, there are no established guidelines for performing episiotomy, and the decision to carry out the procedure rests on the clinical judgment of the attending accoucheur. Thus, it is crucial to assess the current rates of episiotomy and identify the associated risk factors. Previous research has indicated that episiotomy is linked to various maternal sociodemographic characteristics, fetal conditions, and obstetric factors. However, the influence of factors such as the duration of the second stage of labor, the type of childbirth attendant, gestational age, maternal age, and oxytocin use remains controversial.

The systematic identification and quantification of risk factors for episiotomy enable better decision-making and encourage a decrease in episiotomy rates, according to WHO guidelines. Therefore, this systematic review aimed to identify risk factors for episiotomy during vaginal birth.

## 2. Materials and methods

The review was conducted following the Preferred Reporting Items for Systematic Reviews and Meta-Analyses (PRISMA) guidelines.^[13]^ Additionally, the prospective review has been registered with PROSPERO (International Prospective Register of Systematic Reviews) under the registration number CRD420251023510.

### 2.1. Search strategy and selection criteria

A systematic search was conducted across 7 databases, including PubMed/MedLine, Embase, Cochrane, Web of Science, Chinese Knowledge Infrastructure (CNKI), and Wanfang, up to January 2025. Initially, all articles published in English or Chinese were included in the analysis. The research focused on the keywords: “vaginal delivery” [MeSH Terms] OR “vaginal birth” [MeSH Terms]) AND (“episiotomy” [MeSH Terms] OR “perineal incision” [MeSH Terms]) AND (“risk factors” [MeSH Terms] OR “influencing factors” [MeSH Terms].

### 2.2. Inclusion and exclusion criteria

The inclusion criteria were: Studies including both episiotomy and non-episiotomy groups with intergroup comparisons, studies reporting specific odds ratios (OR) for risk factors associated with episiotomy, and studies adopting a paired cohort or case-control design.

The exclusion criteria were: Studies that do not meet the inclusion requirements, such as unpublished articles, reviews, conference reports, expert presentations, master’s and doctoral theses, case reports, and literature review articles; similar studies published by the same authors from the same institution; only the most comprehensive study with relevant information will be retained; literature that presents qualitative, semiquantitative, or nonquantitative analysis methods; research with a sample size of fewer than 20 cases; studies with only abstracts or full texts that cannot be accessed; and animal studies.

### 2.3. Data extraction

Literature screening and data extraction were performed independently by 2 researchers, who then verified each other’s findings. Data were systematically collected from the eligible studies using a standardized form, which included the following information: authors, publication year, country, age, gestational week, sample size, and identified risk factors for episiotomy.

## 3. Quality assessment

Three researchers conducted a thorough assessment of the quality of the selected studies. The quality of the included randomized controlled trials (RCTs) was assessed using a modified Jadad scale, which has a maximum score of 7 points, categorizing studies as low quality (1–3 points) or high quality (4–7 points).^[14]^ For non-randomized controlled trials (non-RCTs), the assessment was performed using the Methodological Index for Non-Randomized Studies (MINORS) rating scale, which has a scoring range of 0 to 24, with classifications as low quality (0–12 points), moderate quality (13–18 points), and high quality (19–24 points).^[15]^

### 3.1. Statistical analysis

Statistical analyses were carried out using Stata 18.0 (StataCorp), while Review Manager 5.4 was used for validating the results. To assess heterogeneity, we applied the *I*^2^ statistic and Cochran *Q* test, selecting the appropriate analytical model based on the level of heterogeneity: a random effects model for *P* < .10 and *I*^2^ > 50% (indicating significant heterogeneity) and a fixed effects model for *P* ≥ .10 and *I*^2^ ≤ 50% (indicating no significant heterogeneity). We used the Egger test and funnel plots to evaluate publication bias when 4 or more articles were included in the analysis. Additionally, meta-regression and subgroup analyses were performed to explore the potential impact of non-research factors on the results. *P* < .05 indicated that the difference was statistically significant.

## 4. Results

### 4.1. Search strategy

**Figure 1.**
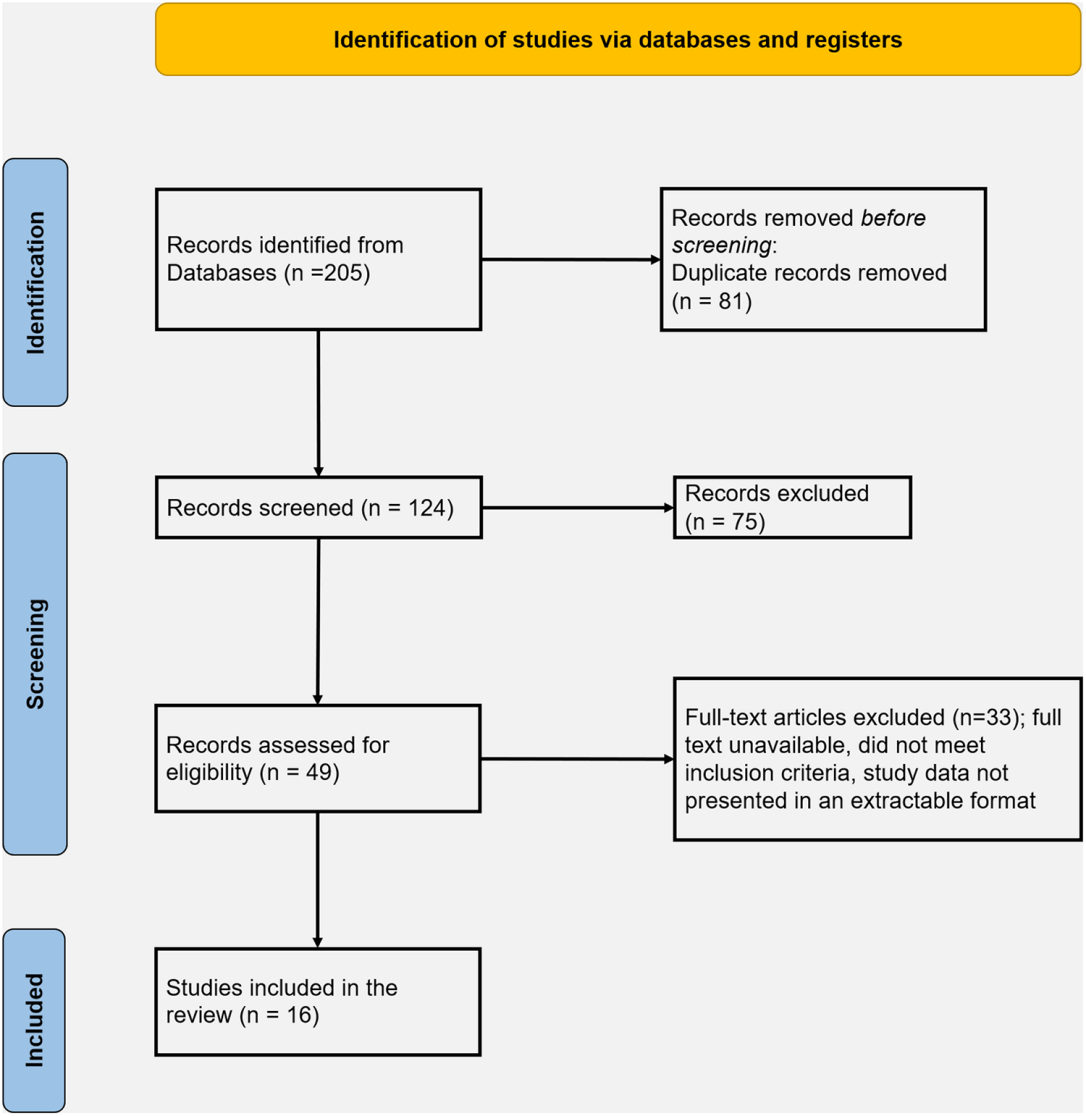
Flow diagram of study selection.

A total of 205 records were obtained from the database search. After eliminating 81 duplicates, 124 records were discarded based on the screening of titles and abstracts. Subsequently, an additional 75 records were excluded following a thorough review of the full texts. Finally, 16 studies satisfied all the stringent criteria outlined in our research protocol and were included in the final meta-analysis. The PRISMA flow diagram is presented in Figure 1, while a comprehensive characterization of the included studies is detailed in Table 1.

**Table 1:** Characteristics of included studies.

Author	Year	Sample size		Maternal age (yr)	Gestational age (wk)	Risk factors				
		Experimental	Control			Age of newborn	Childbirth attendant	Duration of second-stage labor	Weight newborn	Oxytocin use
Gu et al^[16]^	2024	109	40	NR	39.9 ± 0.89	NR	2.708 (1.141–6.425)	0.138 (0.043–0.440)	NR	NR
Zhang et al^[17]^	2024	207	421	NR	NR	1.799 (0.804–4.026)	0.946 (0.594–1.508)	1.359 (0.822–2.246)	NR	3.291 (1.494–7.250)
Zhang et al^[18]^	2022	1292	700	NR	35 ± 7	1.864 (0.918~3.786)	NR	0.194 (0.113~0.334)	NR	NR
Chen et al^[19]^	2021	200	200	NR	NR	NR	NR	4.514 (2.695–7.562)	NR	NR
Xie et al^[20]^	2017	120	120	24.05 ± 4.64	39.26 ± 1.34	0.954 (0.882–1.033)	NR	0.602 (0.515–0.704)	NR	NR
Hu et al^[21]^	2017	2898	6302	26.4 ± 4.4	39.0 ± 1.0	NR	NR	1.774 (1.222–2.117)	1.880 (1.207–2.235)	NR
Xu et al^[22]^	2022	72	173	27.24 ± 3.56	NR	0.97 (0.88–1.06)	NR	1.03 (1.01–1.04)	0.88 (0.42–1.83)	NR
Beyene et al^[23]^	2020	169	242	26.26 ± 4.66	NR	1.21 (0.548–2.812)	NR	1.647 (0.92–2.94)	2.50 (0.81–7.77)	2.60 (1.43–4.75)
Bekele et al^[24]^	2022	177	231	25.10 ± 4.30	NR	NR	NR	1.20 (0.80–2.10)	NR	NR
Woretaw et al^[25]^	2021	181	229	NR	NR	NR	NR	NR	NR	2.73 (1.19–6.25)
Worku et al^[26]^	2019	134	247	27.70 ± 4.20	NR	NR	NR	1.340 (0.518–3.465)	2.857 (1.232–6.625)	NR
Fikadu et al^[27]^	2020	272	128	NR	NR	NR	NR	NR	4.80 (2.70–8.80)	NR
Tefera et al^[28]^	2019	265	140	25.00 ± 4.94	NR	NR	NR	7.60 (5.90–8.60)	4.10 (1.00–6.40)	NR
Teshome et al^[29]^	2020	146	160	NR	NR	1.65 (1.02–2.66)	0.56 (0.47–3.33)		2.48 (1.16–5.31)	NR
Pebolo et al^[30]^	2019	181	68	NR	NR	1.1 (0.499–2.419)	0.9 (0.349–2.098)	3.6 (1.656–7.860)	2.1 (0.655–6.802)	NR
Innocent et al^[31^^]^	2018	939	939	NR	NR	NR	0.6 (0.32–0.85)	NR	NR	1.5 (0.98–3.58)

NR = not reported.

### 4.2. Characteristics of the included studies

A total of 16 articles were included in the meta-analysis, comprising a sample size of 17,502 individuals (7362 cases and 10,340 controls), with studies conducted between 2017 and 2024. Among these studies, the following risk factors for episiotomy during vaginal delivery were identified: newborn age was examined in 7 studies, the attending childbirth provider in 5 studies, the duration of the second stage of labor in 12 studies, newborn weight in 8 studies, and oxytocin usage in 4 studies. The MINORS score of the 16 articles included in this study was ≥20 points, and all of them were high-quality articles.^[16-31]^

### 4.3. Maternal age

**Figure 2.**
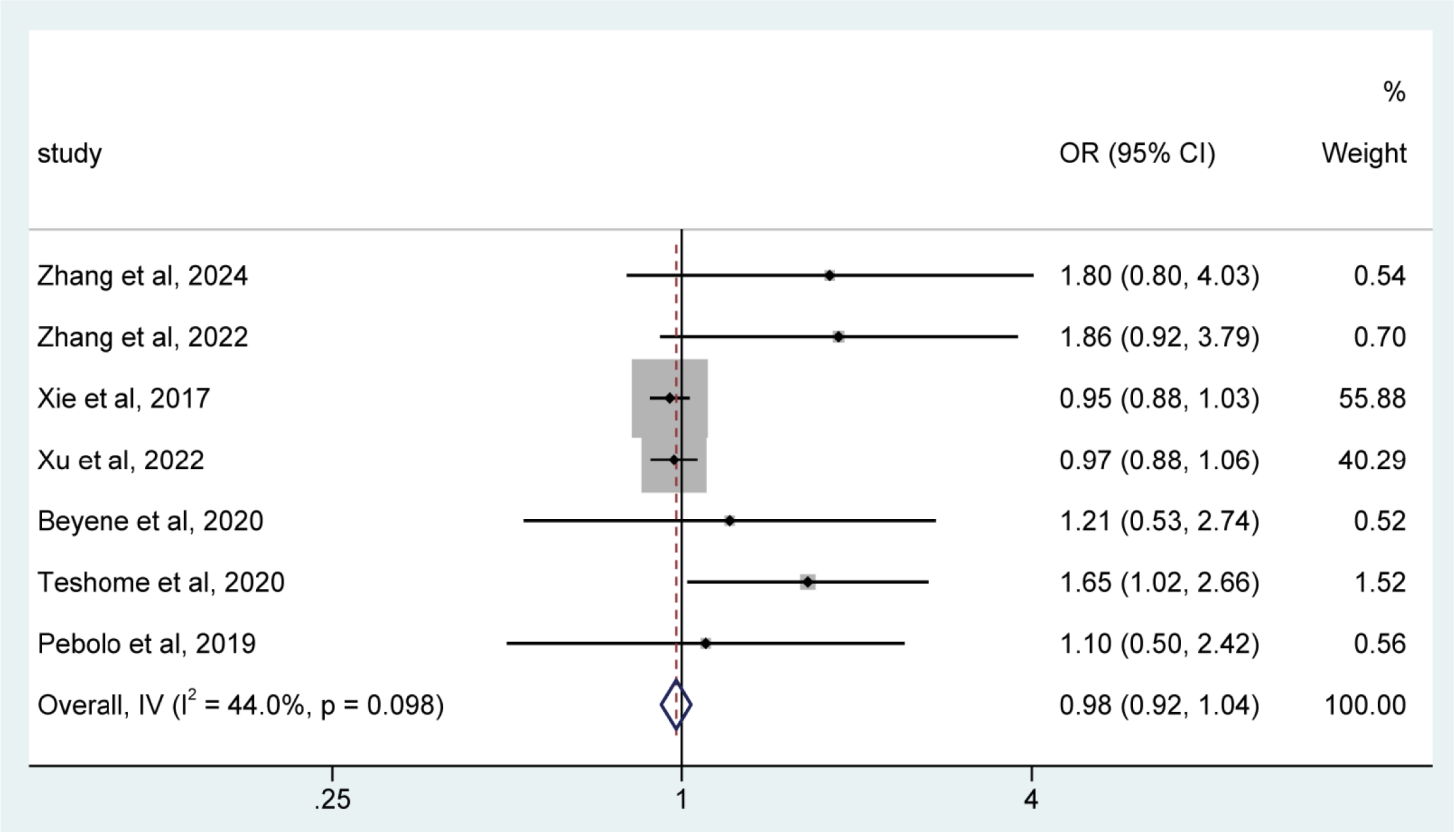
Forest plot illustrating the association between maternal age and episiotomy. CI = confidence interval, IV = inverse variance, OR = odds ratio.

Data from 7 studies examining the impact of maternal age on the incidence of episiotomy were included in the meta-analysis. The findings indicated that maternal age is not a significant risk factor for episiotomy (OR = 0.978; 95% confidence interval [CI] = 0.922–1.038; *P* = .466). Heterogeneity analysis revealed no significant heterogeneity among the studies (Cochran *Q*^2^ = 10.71, *I*^2^ = 74.8%, *P* = .098; Fig. 2). The sensitivity analysis indicated that the outcomes of this meta-analysis were stable and reliable. Egger test indicated the possibility of publication bias (*P* = .014), and a visual examination of the funnel plot corroborated the observed asymmetry, as shown in Figure S1, Supplemental Digital Content 1.

### 4.4. Type of childbirth attendant

**Figure 3.**
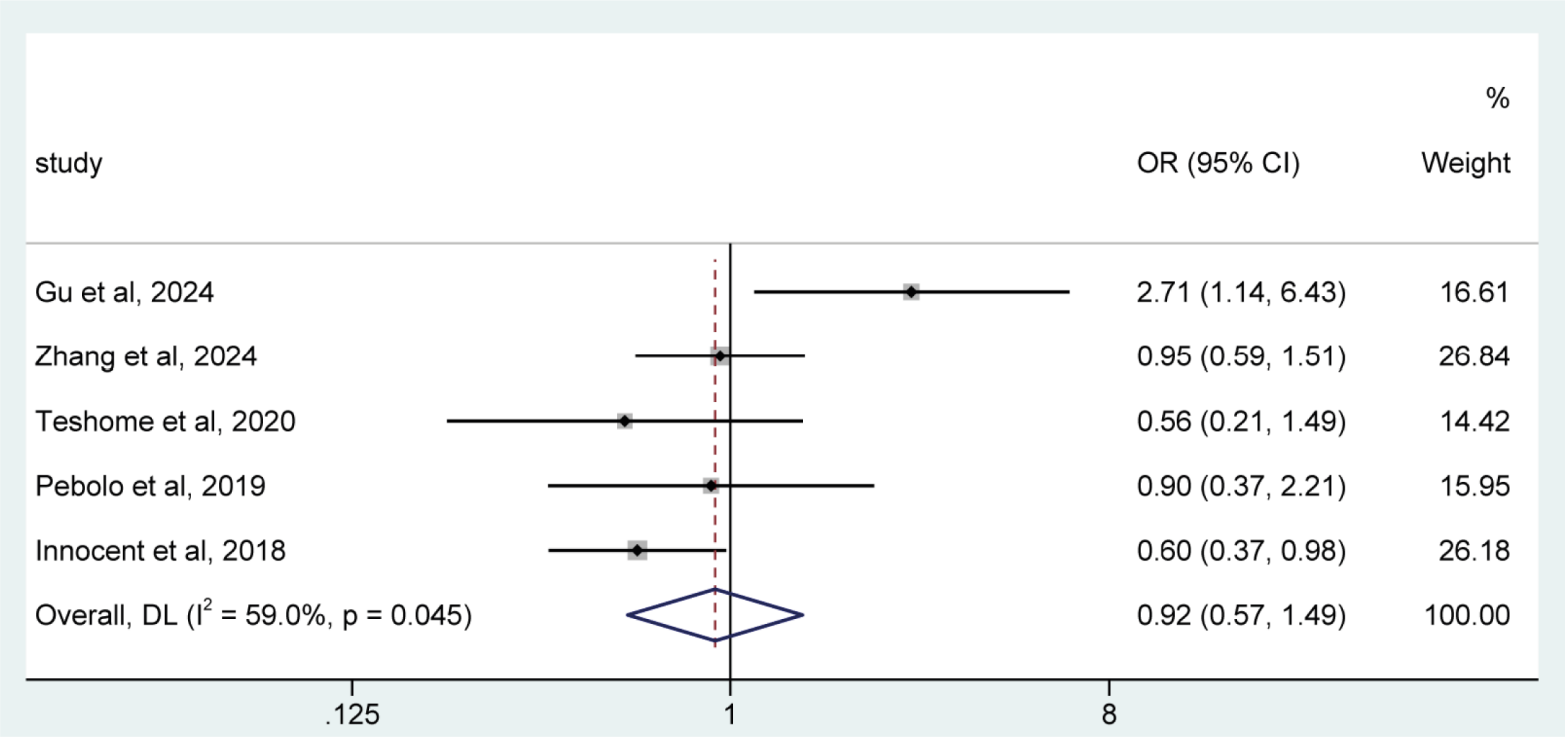
Forest plot illustrating the association between the type of childbirth attendant and episiotomy. CI = confidence interval, IV = inverse variance, OR = odds ratio.

Data from 5 studies assessing the influence of the type of childbirth attendants on the rate of episiotomy were incorporated into the meta-analysis. The results demonstrated that the type of childbirth attendant is not a significant risk factor for episiotomy (OR = 0.920; 95% CI = 0.569–1.488; *P* = .733). Heterogeneity analysis showed substantial variation among the studies (Cochran *Q*^2^ = 9.76, *I*^2^ = 85.9%, *P* = .045; Fig. 3). Furthermore, the sensitivity analysis confirmed the stability and reliability of the meta-analysis results. Egger tests suggested that there was no evidence of publication bias (*P* = .593), and visual inspection of the funnel plot confirmed the absence of asymmetry, as shown in Figure S2, Supplemental Digital Content 2.

### 4.5. Duration of second-stage labor

**Figure 4.**
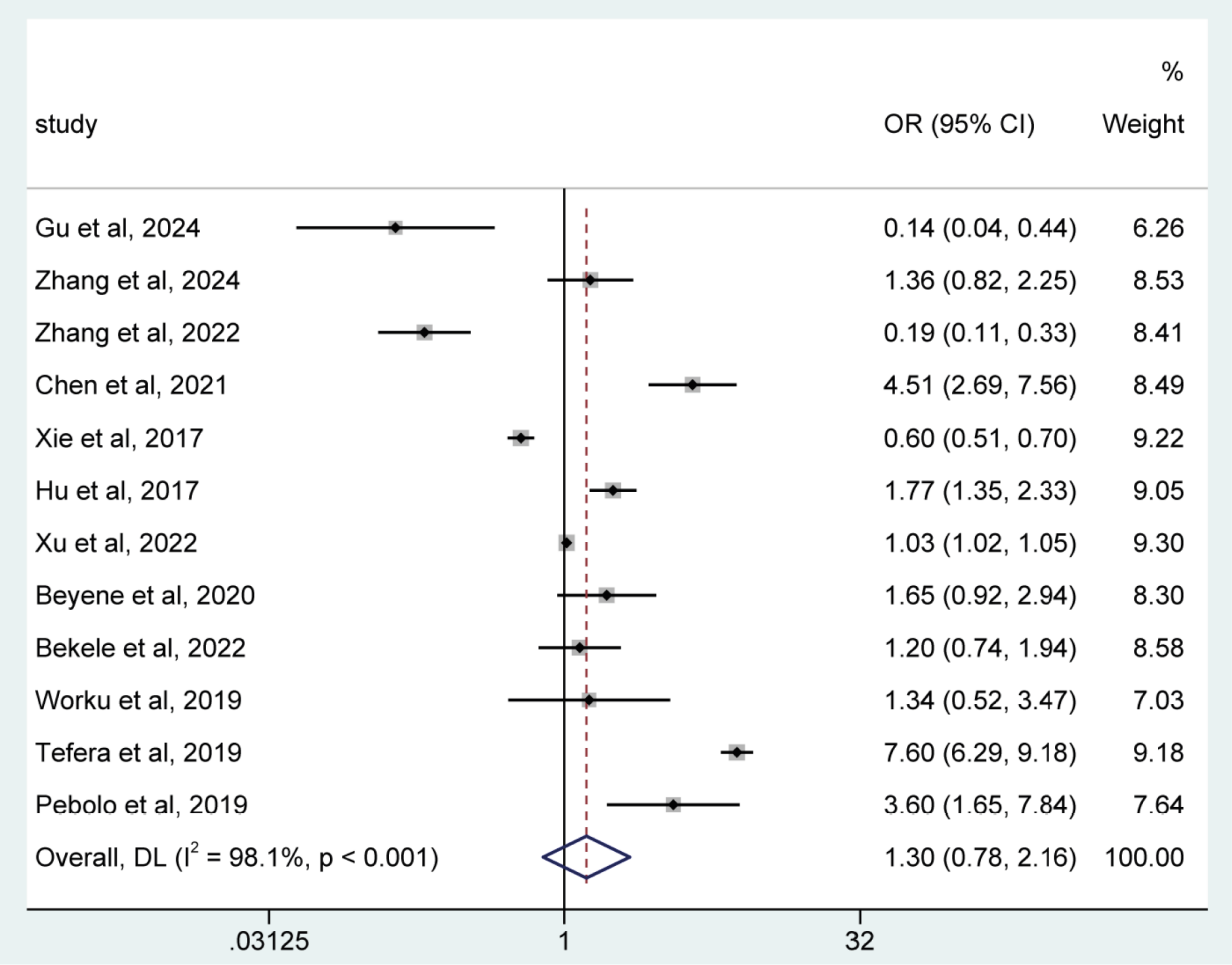
Forest plot illustrating the association between the duration of the second-stage labor and episiotomy. CI = confidence interval, IV = inverse variance, OR = odds ratio.

Data from 12 studies assessing the influence of the duration of second-stage labor on the rate of episiotomy were incorporated into the meta-analysis. The results demonstrated that the duration of second-stage labor is not a significant risk factor for episiotomy (OR = 1.299; 95% CI = 0.781–2.162; *P* = .314). Heterogeneity analysis showed substantial variation among the studies (Cochran *Q*^2^ = 584.76, *I*^2^ = 99.5%, *P* < .001; Fig. 4). Furthermore, the sensitivity analysis confirmed the stability and reliability of the meta-analysis results. Egger tests suggested that there was no evidence of publication bias (*P* = .430), and visual inspection of the funnel plot confirmed the absence of asymmetry, as shown in Figure S3, Supplemental Digital Content 3.

### 4.6. Weight of newborn

**Figure 5.**
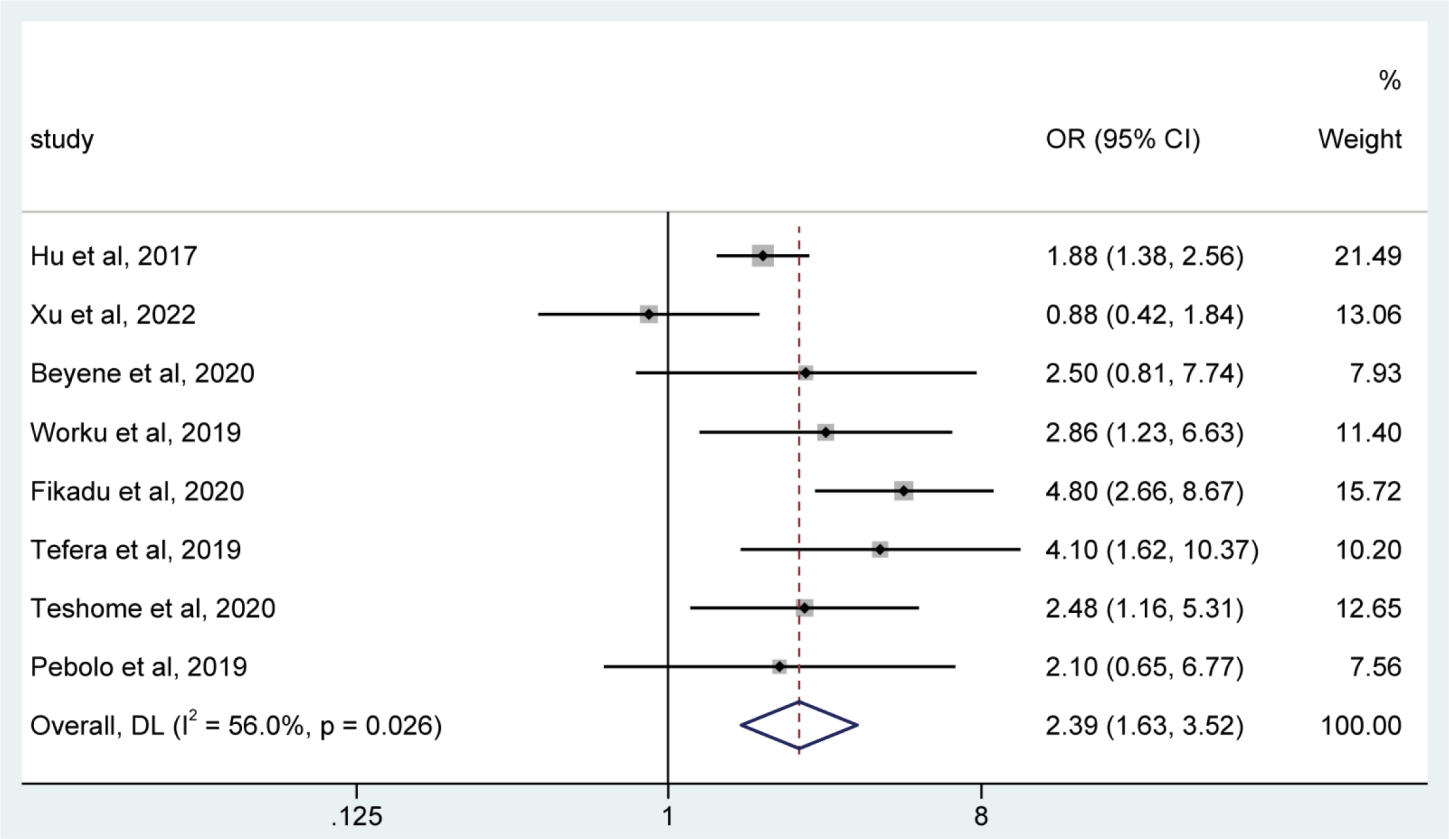
Forest plot illustrating the association between the weight of the newborn and episiotomy. CI = confidence interval, IV = inverse variance, OR = odds ratio.

Data from 8 studies evaluating the effect of newborn weight on the incidence of episiotomy were included in the meta-analysis. The findings indicated that newborn weight is a significant risk factor for episiotomy (OR = 2.393; 95% CI = 1.627–3.520; *P* < .001). Heterogeneity analysis revealed considerable variability among the studies (Cochran *Q*^2^ = 15.89, *I*^2^ = 82.0%, *P* = .026; Fig. 5). Additionally, the sensitivity analysis confirmed the robustness and reliability of the meta-analysis results. Egger tests suggested that there was no evidence of publication bias (*P* = .553), and visual inspection of the funnel plot confirmed the absence of asymmetry, as shown in Figure S4, Supplemental Digital Content 4.

### 4.7. Oxytocin use

**Figure 6.**
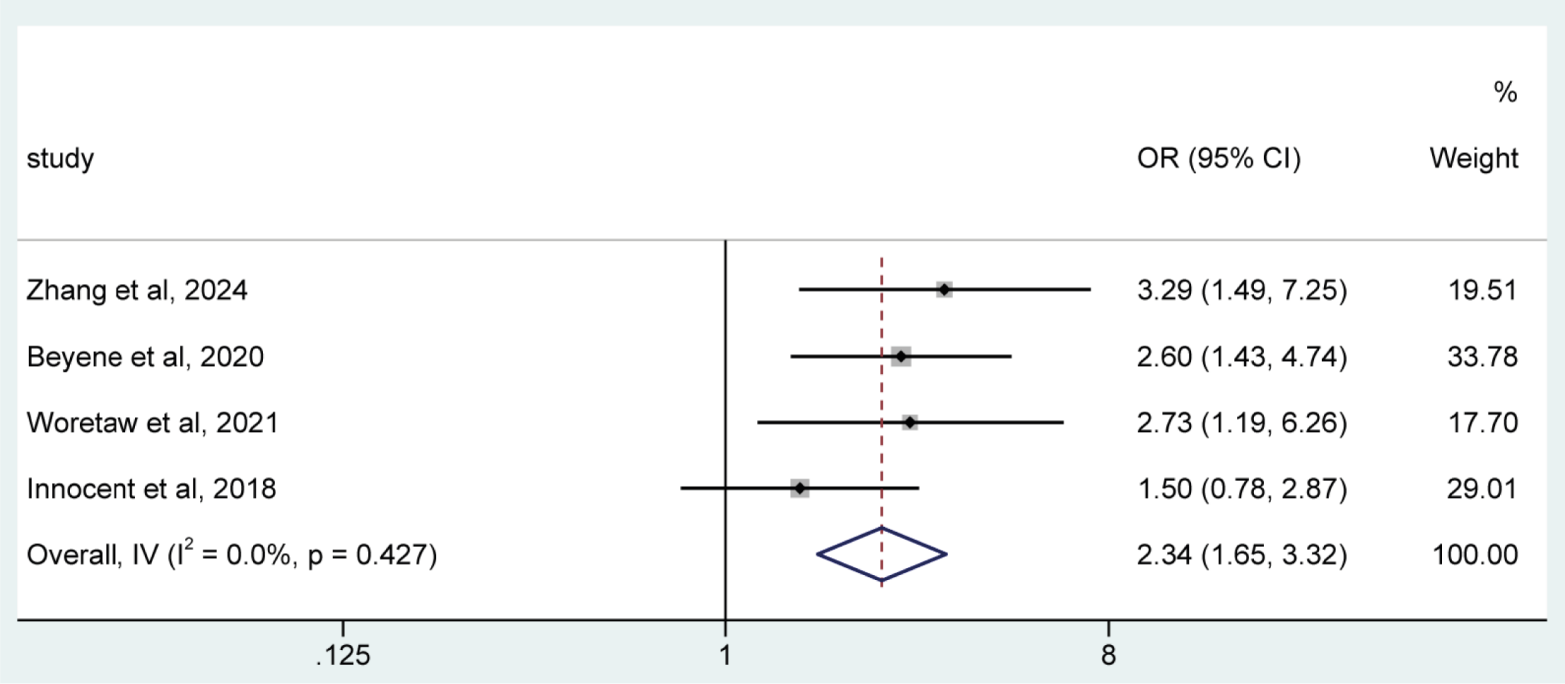
Forest plot illustrating the association between oxytocin use and episiotomy. CI = confidence interval, IV = inverse variance, OR = odds ratio.

Data from 8 studies evaluating the effect of oxytocin use on the incidence of episiotomy were included in the meta-analysis. The findings indicated that oxytocin use is a significant risk factor for episiotomy (OR = 2.341; 95% CI = 1.652–3.318; *P* < .001). Heterogeneity analysis revealed considerable variability among the studies (Cochran *Q*^2^ = 2.78, *I*^2^ = 67.9%, *P* = .427; Fig. 6). Additionally, the sensitivity analysis confirmed the robustness and reliability of the meta-analysis results. Egger tests suggested that there was no evidence of publication bias (*P* = .539), and visual inspection of the funnel plot confirmed the absence of asymmetry, as shown in Figure S5, Supplemental Digital Content 5.

## 5. Discussion

The results of our study indicate that high neonatal birth weight and the administration of oxytocin are associated with an increased risk of episiotomy during delivery. Conversely, maternal age, the type of childbirth attendant, and the duration of the second stage of labor were not associated with an increased risk of episiotomy.

Episiotomy can lead to various complications, including increased bleeding, infection, wound dehiscence, hematoma formation, perineal pain, and the risk of extensive tearing of the anal and rectal sphincters.^[32,33]^ Additionally, long-term complications may arise, such as dyspareunia, anorectal dysfunction, and sexual dysfunction.^[34,35]^ Recent studies show that 40.79% of women who underwent episiotomy were unaware of the procedure, and many also experienced cesarean sections, induction of labor, and vaginal examinations without providing informed consent.^[36]^

Our study showed that higher newborn birth weight significantly increases the risk of needing an episiotomy. This finding aligns with other research indicating that a birth weight of ≥4 kg heightens the likelihood of an episiotomy.^[23]^ In particular, 1 study noted a tendency to avoid episiotomies for newborns weighing <2.5 kg, while those weighing between 2.5–4 kg and over 4 kg were more likely to receive the procedure.^[37]^ Moreover, it was found that for every additional 100 grams of birth weight, the relative odds of undergoing an episiotomy increased by 5.4% overall and by 6.1% among primiparas.^[38]^ Heavier birth weights are often associated with larger fetal head sizes, which can complicate passage through the perineal body and lead to episiotomies aimed at expanding the vaginal opening. Additionally, other studies have indicated a clear link between birth weights >4 kg and a higher frequency of episiotomy.^[39,40]^

The decision to perform an episiotomy is affected by various factors in each context, including social, economic, and cultural backgrounds; the distinction between private and public institutions; differences in models of care (midwifery-led vs physician-led); and the awareness of potential lawsuits. Various studies have indicated an increased risk of episiotomy associated with the use of oxytocin during labor. Specifically, research has shown that women who received oxytocin were 2.73 times more likely to require a perineal incision compared to those who did not receive the hormone.^[25]^ This heightened risk may be linked to the potential for excessive uterine contractions caused by improper administration of oxytocin, which can adversely affect fetal heart rate.^[25]^ Furthermore, the use of oxytocin may disrupt the coordination of contractions in the uterine and pelvic floor muscles, potentially leading to a prolonged second stage of labor.^[41]^ This, in turn, contributes to an increased likelihood of episiotomy. Our research corroborates these findings, revealing that the administration of oxytocin during the second stage of labor is associated with a greater risk of episiotomy during vaginal birth.

No association was found between episiotomy and maternal age, the type of childbirth attendant, or the duration of the second stage of labor. This suggests that while these factors may increase the risk of a cesarean section, they do not seem to influence the clinical decision to perform an episiotomy.

This meta-analysis presents several limitations: the literature included primarily consisted of studies published in English and Chinese, which may introduce a language bias; there was considerable variation in the quality of the randomized controlled trials (RCTs) included, with relatively few studies of high quality, potentially affecting the overall evaluation of episiotomy risk factors during vaginal delivery; although sensitivity analyses and assessments for publication bias were conducted, it remains challenging to eliminate the effects of potential biases and confounding factors.

## 6. Conclusions

Our study indicates that high newborn weight and the use of oxytocin are associated with higher rates of episiotomy during vaginal birth. Professional training, internal audits, and local clinical guidelines are needed to curb the overuse of episiotomy.

## Author contributions

**Conceptualization:** Xuemei He.

**Data curation:** Xuemei He, Demei Lu, Jinping Feng.

**Formal analysis:** Xuemei He.

**Funding acquisition:** Jinping Feng.

**Investigation:** Demei Lu.

**Methodology:** Xuemei He, Demei Lu, Ruifeng Ou.

**Resources:** Jinping Feng.

**Supervision:** Jinping Feng.

**Validation:** Ruifeng Ou, Jinping Feng.

**Visualization:** Ruifeng Ou.

**Writing – original draft:** Xuemei He, Demei Lu, Ruifeng Ou, Jinping Feng.

**Writing – review & editing:** Xuemei He, Demei Lu, Ruifeng Ou, Jinping Feng.










