# Supplementary material for: Risk factors for episiotomy during vaginal birth: A systematic review and meta-analysis
Source: Medicine (Baltimore). 2026 Jul 10;105(28):e49662. doi: 10.1097/MD.0000000000049662 (PMC13362934; doi:10.1097/MD.0000000000049662)
Supplement: Supplementary file 3 [file medi-105-e49662-s003.docx]

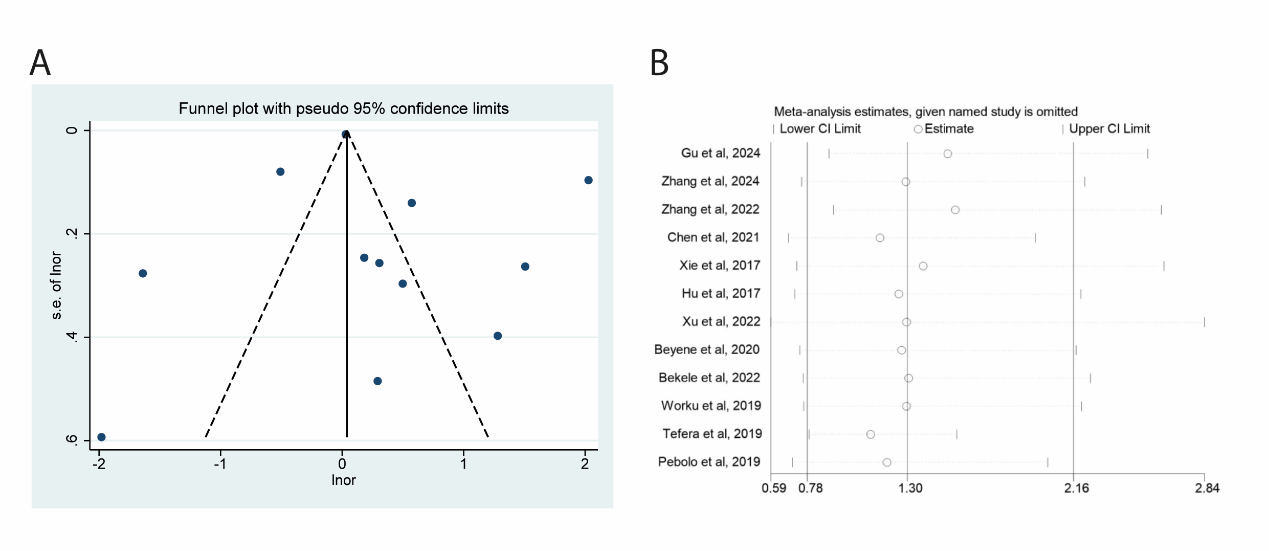


Figure S3. Publication bias assessment and sensitivity analysis for the association between the duration of second-stage labor and episiotomy.
